# Selective auditory grouping by zebra finches: testing the iambic–trochaic law

**DOI:** 10.1007/s10071-017-1089-3

**Published:** 2017-04-08

**Authors:** Michelle Spierings, Jeroen Hubert, Carel ten Cate

**Affiliations:** 1 Behavioural Biology, Institute of Biology Leiden (IBL), Leiden University, P.O. Box 9505, 2300 RA Leiden, The Netherlands; 2 Leiden Institute for Brain and Cognition (LIBC), Leiden University, P.O. Box 9600, 2300 RC Leiden, The Netherlands

**Keywords:** Iambic/trochaic law (ITL), Perceptual bias, Acoustic perception, Zebra finches

## Abstract

Humans have a strong tendency to spontaneously group visual or auditory stimuli together in larger patterns. One of these perceptual grouping biases is formulated as the iambic/trochaic law, where humans group successive tones alternating in pitch and intensity as trochees (high–low and loud–soft) and alternating in duration as iambs (short–long). The grouping of alternations in pitch and intensity into trochees is a human universal and is also present in one non-human animal species, rats. The perceptual grouping of sounds alternating in duration seems to be affected by native language in humans and has so far not been found among animals. In the current study, we explore to which extent these perceptual biases are present in a songbird, the zebra finch. Zebra finches were trained to discriminate between short strings of pure tones organized as iambs and as trochees. One group received tones that alternated in pitch, a second group heard tones alternating in duration, and for a third group, tones alternated in intensity. Those zebra finches that showed sustained correct discrimination were next tested with longer, ambiguous strings of alternating sounds. The zebra finches in the pitch condition categorized ambiguous strings of alternating tones as trochees, similar to humans. However, most of the zebra finches in the duration and intensity condition did not learn to discriminate between training stimuli organized as iambs and trochees. This study shows that the perceptual bias to group tones alternating in pitch as trochees is not specific to humans and rats, but may be more widespread among animals.

## Introduction

When hearing a long string of successive tones that alternate in pitch, intensity or duration, humans tend to perceive them as a concatenation of duplets that either have prominence on the first tone (trochees) or prominence on the second tone (iambs). When the tones are alternating in pitch or intensity, they are grouped as trochees and tones alternating in duration are often, but not universally, grouped as iambs (Hayes 1985, 1995; Hay and Diehl 2007; Bion et al. 2011). This grouping principle, known as the iambic–trochaic law (ITL), has been known for over a century (Bolton 1894; Woodrow 1909) and has been confirmed by numerous studies (Vos 1977; Hayes 1985, 1995; Crowhurst and Olivares 2014).

In behavioural paradigms, this perceptual ability and grouping bias becomes clear around 8 months of age, when English-speaking infants segment strings of tones alternating in intensity as trochees and alternating in duration as iambs (Trainor and Adams 2000). The early onset of the ITL strengthened the idea that this might be a universal principle that is shared between different age classes. Another indicator of the ITL as a universal grouping principle is the fact that the perceptual grouping is not restricted to a particular sound type: human adults and infants show perceptual grouping of musical tones, beeps or spoken syllables (e.g. Monahan and Carterette 1985; Hay and Diehl 2007; Iversen et al. 2008; Bhatara et al. 2015; Crowhurst and Olivares 2014). Lastly, there is great similarity between the principles of the ITL and the Gestalt principles applying to perception of visual objects, also arguing for a universal perceptual principle (for a review, see Wagemans et al. 2012).

In language perception, the ITL plays a role in the perception of words and the segmentation of speech streams (Bion et al. 2011; Hay and Saffran 2012). For example, in English 90% of words have a trochaic stress pattern (Cutler and Carter 1987). In line with this, English infants of 7.5 months old are better at recognizing trochees in a string of continuous speech sounds (Cutler and Carter 1987; Jusczyk et al. 1999) and 9-month-old infants already have a general preference for listening to trochees over iambs (Jusczyk et al. 1993). From 5 months of age onwards, infants show an increased brain response to trochees in a string of iambs and have a preference for the iambic or trochaic stress pattern of their native language (Weber et al. 2004; Friederici et al. 2007). This means that they have a perceptual grouping bias from an early age onwards, which may be affected by acoustic experience at an early age. Even learning a second language does not change this perceptual bias (Bijeljac-Babic et al. 2016). Furthermore, when infants are faced with the task of segmenting a string of speech sounds into words, they use the natural stress pattern of their native language as a clue for word boundaries (Morgan 1996; Thiessen and Saffran 2003, 2007). These examples show that perceptual grouping biases play an important role in speech processing and that there might be an influence of the native language of the listener on the development of these grouping biases. However, in most cross-linguistic studies with adult or infant participants, they group alternations in pitch and intensity as trochees, independent of the rhythmic pattern of their native language (e.g. Iversen et al. 2008; Molnar et al. 2014, 2016, but see Bhatara et al. 2015). The perceptual grouping of alternations in duration seems more strongly affected by early acoustic experience. For example, in line with their language pattern, native speakers of English and German group alternations in duration as iambs (Hay and Diehl 2007; Höhle et al. 2009; Bhatara et al. 2015; Segal and Kishon-Rabin 2012; Crowhurst 2013), whilst Zapotec and Japanese speakers, both adults and infants, group these alternations as trochees (Yoshida et al. 2010; Crowhurst and Olivares 2014). These studies show that there is a clear effect of acoustic experience on the perception of alternations in duration, but that the perception of alternations in pitch and intensity might be more universal.

The strong tendency for perceptual grouping present in young infants, and the strong bias to group pitch and intensity alternations as trochees irrespective of linguistic experience raise the question whether non-human animals also show perceptual grouping, and if so, whether they show similar biases to humans. A first indication that it may be a more general perceptual phenomenon is that rats also group tones alternating in pitch as trochees (de la Mora et al. 2013). The rats were trained to discriminate tonal strings alternating in pitch or duration from strings in which the tones were randomly organized. They only received food for pressing a lever after hearing the alternating strings. After they learned to discriminate, they were exposed to pairs of tones, either iambs or trochees. Their lever presses revealed that the rats grouped the pitch-alternating strings as trochees and did not group the tones in the duration-alternating strings (de la Mora et al. 2013). A follow-up showed that when rats were passively exposed to either iambic or trochaic stress patterns, they would group duration-alternating strings in accordance with the pattern they were exposed to (Toro and Nespor 2015). Thus, similar to humans, acoustic experience influenced the perceptual grouping bias.

These results from rats suggest that the trochaic grouping bias for alternations in pitch is not specific to language or humans and may be an ancient principle that humans use to organize speech sounds (Toro 2016). However, with no other animal species tested, the generality of the grouping bias is not clear and it might not be shared among a wider range of species.

In the current study, we explore the presence of iambic or trochaic grouping biases for tones alternating in pitch, duration or intensity in a bird species, the zebra finch. Zebra finches, small songbirds, are a well-studied model species for auditory perception (e.g. Kriengwatana et al. 2014; Dent et al. 2016). Also, they are able to perceive stress in human speech and are sensitive to the stress pattern over a string of speech syllables (Spierings and ten Cate 2014), something that has also been demonstrated in Java sparrows (Naoi et al. 2012) and budgerigars (Hoeschele and Fitch 2016). This shows that birds are sensitive to acoustic features that also influence the iambic/trochaic grouping bias in humans and makes birds an excellent group to examine for the presence and direction of grouping biases.

The zebra finches in the current study were trained to discriminate between alternating tones arranged as iambs and trochees, using a go-left/go-right paradigm. These tones varied in pitch for one group of animals, in duration for a second group and in intensity for the third group. After the zebra finches correctly discriminated between the iambic and the trochaic structures, they were tested with long strings of alternating tones, again either alternating in pitch, duration or intensity. If they perceived these alternations as iambic, they were expected to give a similar response as to the trained iambs. If they perceived them as trochaic, they were expected to give a response similar to that of the trochaic training stimuli.

## Methods

### Subjects

Thirty-two zebra finches were tested (16 males and 16 females); 8 were tested in the pitch group, 12 in the duration group, and 12 in the intensity group. All groups had an equal number of males and females within the group. All zebra finches were at least 160 days old at the beginning of the experiment. The animals were bred and reared at the Leiden University animal breeding facility, where they were housed in single sex groups on a 13.5 L:10.5 D schedule at 20–22 °C. Food, water, grit and cuttlebone were available ad libitum. During the experiment, food was used as reinforcement and therefore only available after a correct trial. Food intake was monitored daily, and additional food was provided whenever necessary.

### Apparatus

The experiments took place in individual operant conditioning cages, which were placed in separate sound-attenuated rooms. Each room was illuminated by a fluorescent tube that emitted a daylight spectrum on the same 13.5 L:10.5 D schedule as was used in the breeding facility. A speaker (Vifa 10BGS119/8) was located 1 m above the centre of the cage. The operant conditioning cages were constructed of mesh wire sides with a back wall and floor of foamed PVC. The back wall supported three horizontally aligned pecking keys and a food hatch above them, all easily accessible from various perches. The pecking keys were fitted with red LED lights. Birds needed to peck on the middle key to initiate a trial and stimulus playback. Depending on the nature of the playback, the bird had to either peck on the key on the left or the key on the right within 30 s. A correct response was followed by 8 s of food access (seeds identical to their regular diet), and an incorrect response was followed by 15 s of darkness.

### Stimuli

#### Training

**Fig. 1:**
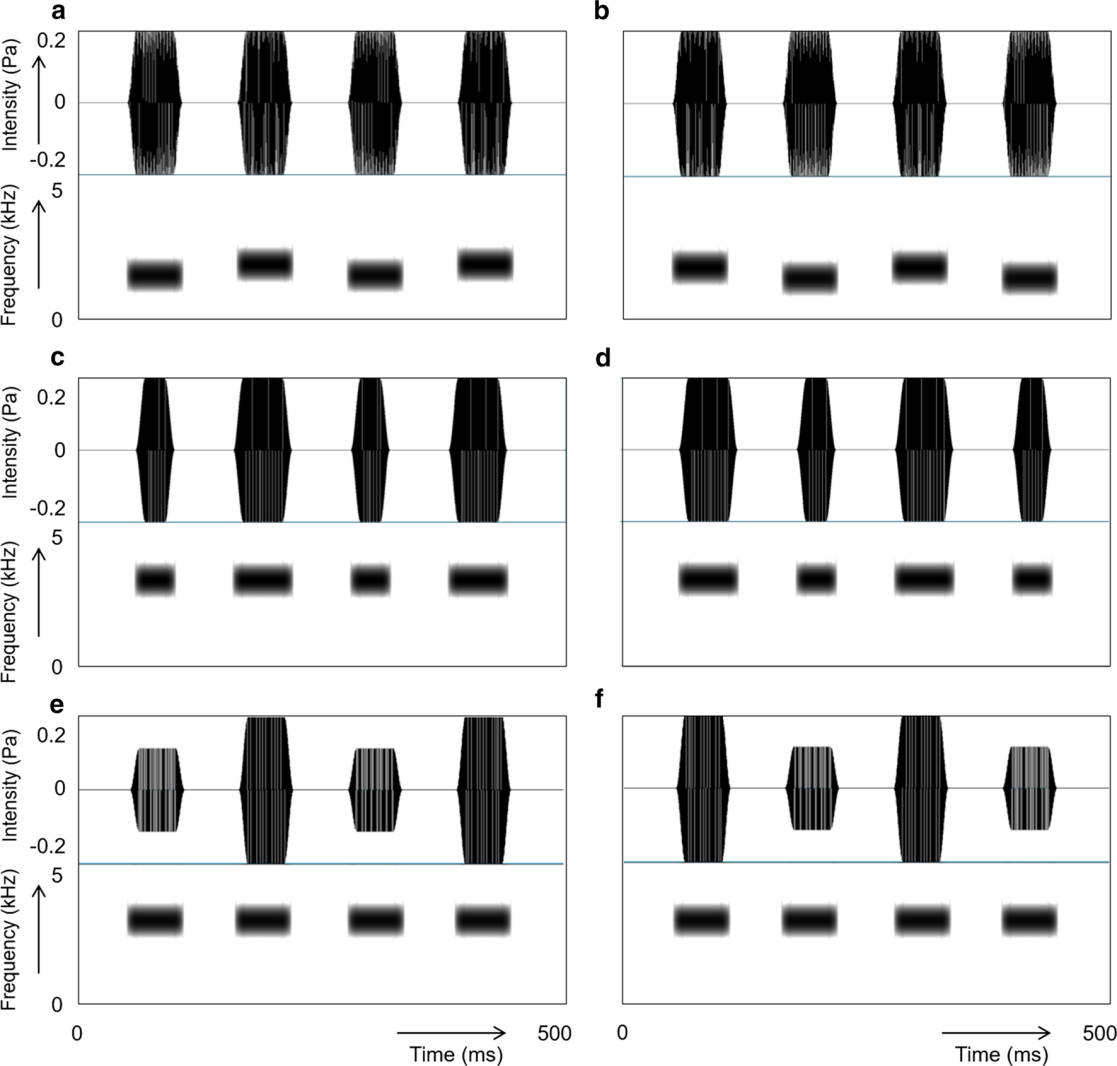
Example of six training stimuli. In each image, the *top part* shows the intensity of the tones and the *bottom half* shows the frequency. Two stimuli have changes in pitch: one stimulus with two duplets with iambic stress (**a**) and one stimulus with two duplets with trochaic stress (**b**); two with changes in duration: duplets with iambic stress (**c**) and duplets with trochaic stress (**d**); and two with changes in intensity: duplets with iambic stress (**e**) and with trochaic stress (**f**)

The birds were trained to discriminate between stimuli that each consisted of two duplets of tones (i.e. four tones organized in an ABAB structure). Half of the stimuli consisted of two duplets with iambic stress, and the other half of two duplets with trochaic stress. For one group of birds, the stress was created by changes in pitch, for a second group by changes in duration, and for a third group by changes in intensity (see Fig. 1 for an example). Each bird received a set of four stimuli with iambic stress and four with trochaic stress. In the pitch condition, the iambic stimuli had a low–high–low–high pattern and the trochaic stimuli a high–low–high–low pattern. The high tones were always 25% higher than the low tones within the same quadruplet. All tones for this condition were 60 ms long and had an intensity of 70 dB. In the duration condition, the stimuli were organized in a similar fashion, the iambic stimuli had a short–long–short–long pattern, and the trochaic stimuli had a long–short–long–short pattern. Each of the four training stimuli within one category (iambs or trochees) started with a different tone duration, and the long tones were 50% longer than the short tones within the same quadruplet. As the initial group of eight birds trained with these stimuli showed very poor learning, we decided to test four additional zebra finches with a stimulus set in which the long tones were 100% longer than the short tones. All tones in this condition had a pitch of 3 kHz and an intensity of 70 dB. In the intensity condition, the iambic stimuli had a soft–loud–soft–loud pattern and the trochaic stimuli a loud–soft–loud–soft pattern. The loud tones were 5 dB louder than the soft tones. Here also the learning during training was poor, and hence, we again added an additional four zebra finches that were trained with stimuli in which the loud tones were 8 dB louder than the soft tones. All tones in this condition were 60 ms long and had an intensity of 3 kHz. In all conditions, the pure tones were separated by a 60-ms silent interval. For each condition, four different training sets were created to avoid pseudoreplication (see Table 1 for an example of the training stimuli). The pitches, durations and intensities of the tones were all chosen to be within the hearing range of the zebra finches. Furthermore, the differences between the tones within each condition have been shown to be audible for the birds (Spierings and ten Cate 2014).

**Table 1:** Example of a set of training stimuli for the pitch condition (a), the duration condition (b) and the intensity condition (c)

(a) Pitch training	Iambic stimuli (tone frequencies in Hz)	Trochaic stimuli (tone frequencies in Hz)
1	1500–1875–1500–1875	1875–1500–1875–1500
2	1875–2344–1875–2344	2344–1875–2344–1875
3	2344–2930–2344–2930	2930–2344–2930–2344
4	2930–3662–2930–3662	3662–2930–3662–2930

(b) Duration training	Iambic stimuli (tone durations in ms)	Trochaic stimuli (tone durations in ms)
1	40–60–40–60	60–40–60–40
2	60–90–60–90	90–60–90–60
3	90–135–90–135	135–90–135–90
4	135–202–135–202	202–135–202–135

(c) Intensity training	Iambic stimuli (tone amplitudes in dB)	Trochaic stimuli (tone amplitudes in dB)
1	47–52–47–52	52–47–52–47
2	52–57–53–57	57–53–57–52
3	57–62–57–62	62–57–62–57
4	62–67–62–67	67–62–67–62

The Table shows the pitch, duration or intensity of the tones used in the training stimuli rounded to the nearest integer. Shown here is one of the sets used, starting with 1500 Hz (pitch condition), 40 ms (duration condition) and 47 dB (intensity condition). The other three sets were created with a longer, higher or louder start tone (1750, 2000 and 2250 Hz; 45, 50 and 55 ms; and 49, 51 and 53 dB). The relative difference between two consecutive tones remained constant

#### Tests

There were four different test conditions, three testing a potential bias of the birds (Tests 1, 2 and 3) and one control condition (Test 4, Table 2). In Tests 1, 2 and 3, the zebra finches heard long sequences of alternating tones. If they perceived these to be organized in an iambic way, they were expected to categorize them as they did with the iambic training stimuli. If they grouped the tones as trochees, they should respond similarly as to the trochaic training stimuli. Test 4 also had long strings, but consisting of one single tone. These strings could not be grouped based on the alternations, which means that non-random responses of the birds would indicate a response preference for one of the keys or a perceptual grouping bias extended to non-alternating sounds.

**Table 2:** Overview of the test stimuli

	Tones in stimuli	Alternating	Number of different strings
Test 1	Same as training tones	Yes	4
Test 2	New, within range of training tones	Yes	2
Test 3	New, outside range of training tones	Yes	4
Test 4	Same as training tones	No	3

All test strings were 26 tones long and had a fade in and fade out of 1.3 s. The three columns show which type of tones was used to create the stimuli, whether these were alternating in pitch, duration or intensity, and how many different strings were presented to the birds in each test condition. Test strings were presented in 20% of the trials when the zebra finch had reached the standard training criterion

More specifically, the stimuli for Test 1 consisted of the same tones as those used for the second and third training quadruplets (see Table 1; “Appendix”), only now in a string of 26 tones. As in the training stimuli, the tones alternated with high–low, long–short or loud–soft configurations. However, unlike the training stimuli, these long strings started and ended with a 1.3-s fade, obscuring how the string started. This prevented the birds from simply comparing the start or end tones of a string to the training stimuli. Moreover, some test strings started with a stressed tone and others with an unstressed tone. All tones were again separated by 60-ms silent intervals. The stimuli for Tests 2 and 3 were constructed similarly to those of Test 1, but now consisted of new tones. These tones were of either a different pitch (for the pitch condition), duration (for the duration condition) or intensity (for the intensity condition) than the tones from the training stimuli. Test 2 had tones within the range of the training tones, and Test 3 consisted of one tone that was on the edge of the range of the training tones and tones that were higher and lower, longer and shorter, or louder and softer than the training tones (see “Appendix”). As in Test 1, these strings were 26 tones long and had a 1.3-s fade in and fade out. Test 4 consisted of three different test strings, all containing one of the three tones that also occurred in the second and third training strings (see “Appendix”). These test strings did not have alternating tones, but had a repetition of a single tone. Like the strings of Tests 1, 2 and 3, they were 26 tones long, separated by 60-ms silent intervals and with a fade in and fade out of 1.3 s. As there were four different training sets for each condition, there were also four test sets per condition to match the stimuli from the training.

### Experimental design

Each zebra finch was first trained on the go-left/go-right design with two unfamiliar zebra finch songs. They received a reward for pecking on the left key after hearing one song, and on the right key after hearing the other song. When they reached a criterion of over 75% correct responses to both songs for three consecutive days, they proceeded to the training.

During training, the zebra finches had to discriminate between four stimuli with iambic stress and four stimuli with trochaic stress by pecking on either the left or the right key after the stimulus was played. For half of the birds, the key for iambs was on the right side of the cage and the key for trochees on the left; for the other half of the birds, this was switched. If an individual only used one of the response keys instead of both, the programme was set to repeat a stimulus that received an incorrect response until the bird gave the correct response. This setting would be on for <24 h, motivating the animal to use both response keys. Training continued until the birds reached a criterion of >75% correct for three consecutive days or when they reached 20,000 trials without having 3 consecutive days with more than 55% correct responses. Those birds that reached the learning criterion then proceeded to the test phase. One bird from the duration condition and one bird from the intensity condition did not reach the criterion, but their performance was above 60% correct for 10 consecutive days. These two birds also proceeded to the test phase.

In the test phase, 20% of the trials were non-reinforced test stimuli, presented in a random order within a test block. The other 80% of the trials remained reinforced training trials. The test items were organized in two sequentially presented test blocks, one with the stimuli of Tests 1, 2 and 3 and the second one with the stimuli of Test 4. A bird moved to the next test block after each test stimulus in the block had been presented 40 times.

### Analysis

The responses of the birds to the training and test stimuli were calculated as proportions of responses to the key for iambs and the key for trochees per stimulus (number of responses/number of trials). It was also possible for the birds not to respond within 30 s of initiating a trial, in which case a “no response” was recorded. The proportion of no responses was calculated as the number of non-responses divided by the number of trials. For the training, we calculated the average responses to all iambic training stimuli and the average response to the trochaic stimuli per bird. For the test, we calculated the average response towards the different stimuli within the test trials of that condition. The three response proportions (iambic, trochaic and no response) always added up to be a hundred percentage per stimulus. These data were analysed with a generalized linear model (glm) with test item (all tests and the training iambic and trochaic stimuli) as fixed effect and the individual as the random measure. Pairwise comparisons were made between the proportions of responses to the iambic and the trochaic key for each test and the two training sets by using a Tukey’s post hoc test, corrected for multiple testing. When there was only one individual tested in a condition, their responses were analysed with a pairwise *t* test between their responses to the iambic and the trochaic stimuli.

## Results

### Training

**Fig. 2:**
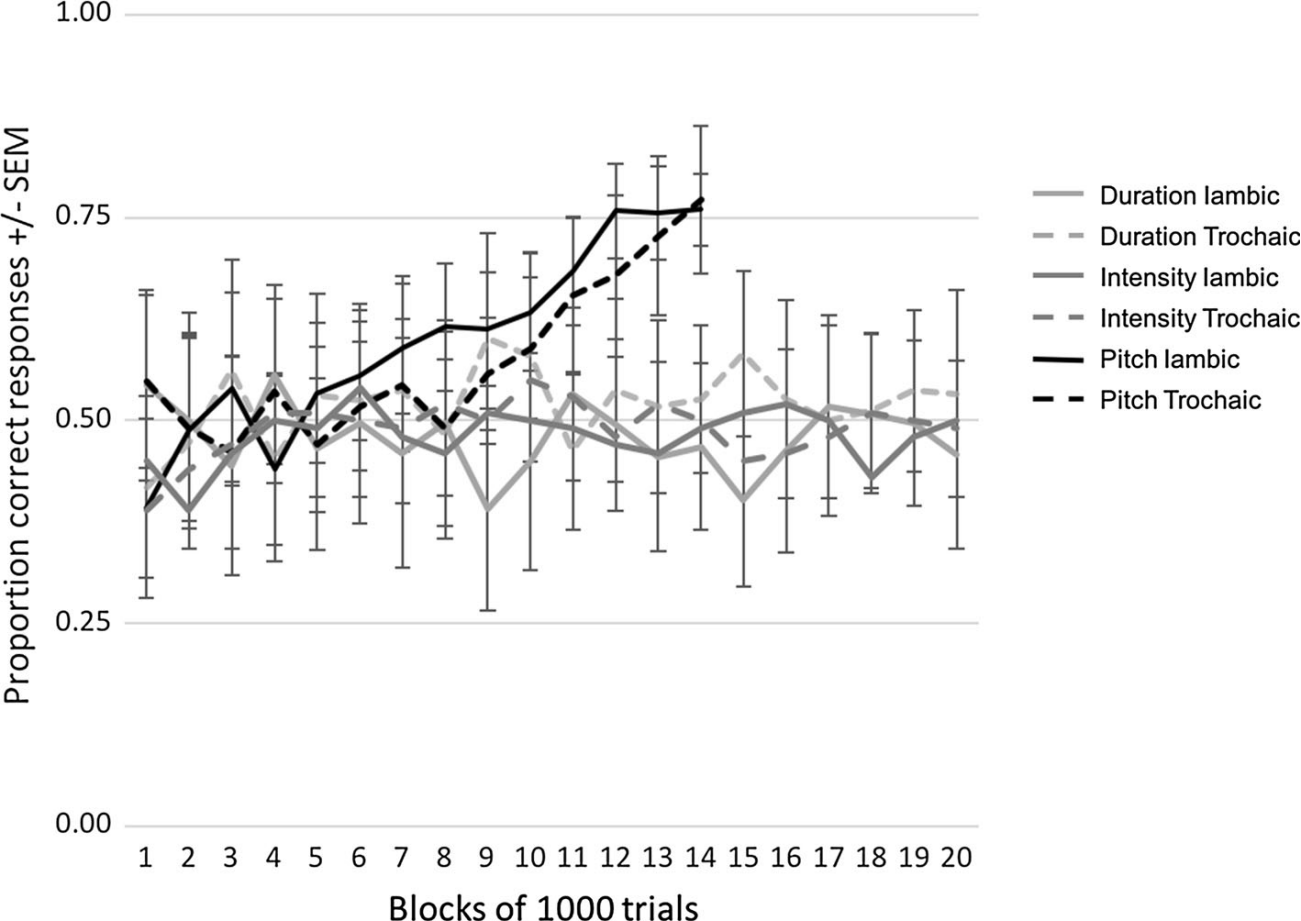
Proportions of correct responses to the iambic and the trochaic training sounds. Duration iambs are quadruplets with increased duration of the second and fourth tone. Duration trochees are quadruplets with increased duration of the first and third tone. In the same fashion, intensity iambs are quadruplets with an increased intensity of the second and fourth tone and intensity trochees have an increased intensity on the first and third tone. Finally, pitch iambs are quadruplets with increased frequency of the second and fourth tone and pitch trochees are quadruplets with increased frequency on the first and third tone. The lines show the average responses of the 8 zebra finches in the pitch condition and 12 zebra finches in both the duration and the intensity condition, organized in blocks of 1000 trials

The training of the birds lasted until they learned the discrimination by reaching a discrimination score of over 75% correct responses to both iambs and trochees for three consecutive days. For the birds that did not learn, the training lasted until they did 20,000 trials without reaching a discrimination score of 55% for 3 consecutive days. If their discrimination scores were higher than 55% at 20,000 trials, they continued the training until they fully learned the discrimination. The zebra finches completed an average of 390 trials a day (SD = 60). All birds in the pitch condition learned the discrimination in <20,000 trials with an average of 14,717 trials (±4118). Their response rate changed from giving a response to, on average, 68% of the trials during the first 500 trials, to giving a response to 93% of the trials during the last 500 trials. In the duration condition with a 50% tone length increase, one of the birds learned the discrimination in a moderate fashion (67% correct responses to iambs and 70% correct to trochees). Also in the intensity condition with a 50% tone intensity increase, one bird learned the discrimination moderately (66% correct responses to iambs and 73% correct to trochees). The other zebra finches were unable to learn the discrimination within the 20,000 trial frame, even when the difference between stressed and unstressed tones was increased to 100% longer (duration condition) or 8 dB louder (intensity condition). The learning curves for all three conditions are shown in Fig. 2.

### Test

**Fig. 3:**
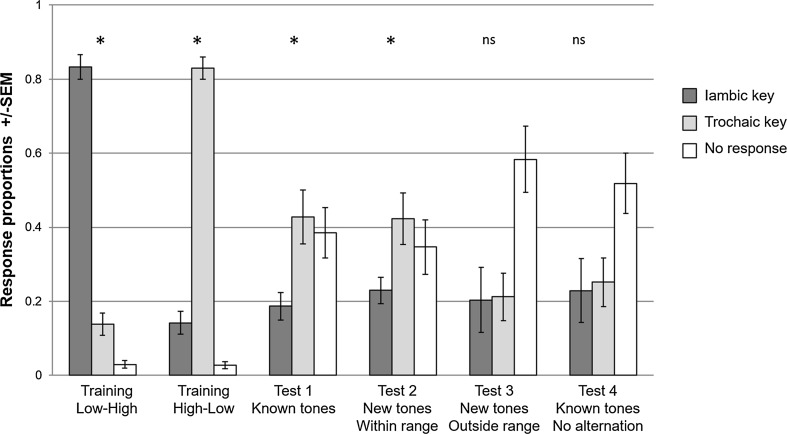
Proportions of responses to the training and test stimuli of the pitch condition. The *dark grey bars* show the proportions of pecks on the iambic key, and the *light grey bars* show the pecks on the trochaic key. The *white bars* show the proportion of trials to which the birds did not respond by pecking on a key. The *bars* show the averages of all 8 zebra finches, and the *error bars* show the SEM

Figure 3 shows the results of the test trials. All zebra finches in the pitch condition reached the discrimination criterion and were tested afterwards. In the pitch test, the zebra finches classified the long alternating strings of known tones in Test 1 more frequently as a trochaic pattern than as an iambic one (mean iambic = 0.19, mean trochaic = 0.43, *P* > 0.01). In 38% of the trials, no pecking response was given. A similar result was found for the strings of Test 2—long alternating strings with new tones within the range of the training tones. The zebra finches showed a difference in responding by classifying the ambiguous stimuli more often as being trochaic than being iambic (mean iambic = 0.23, mean trochaic = 0.42, *P* > 0.01). The zebra finches did not respond in 35% of the trials. When the tones in the long strings were outside the training range (Test 3), the zebra finches did not differentiate (mean iambic = 0.2, mean trochaic = 0.21, *P* = 0.87). The zebra finches responded less to the stimuli of this test, with no pecking response in 58% of the trials. In Test 4, strings without the high–low alternation, the zebra finches also responded equally often by pecking on the iambic as on the trochaic key (mean iambic = 0.23, mean trochaic = 0.25, *P* = 0.64). None of the test conditions showed a different response to the test strings that started with a low tone and test strings that started with a high tone (all *P* > 0.1).

In the duration condition, one bird was tested after reaching a correct proportion of >0.62 for three consecutive days during training. He responded more often to the trochaic than to the iambic key in Tests 1 and 2 (1: mean iambic = 0.16, mean trochaic = 0.34, *P* = 0.02; 2: mean iambic = 0.18, mean trochaic = 0.30, *P* = 0.04). However, when the tones were outside the training range (Test 3) he responded more by pecking on the iambic key (mean iambic = 0.29, mean trochaic = 0.09, *P* = 0.04). In Test 4 (not alternating), he responded more often by pecking on the iambic key than the trochaic key, but the difference was not significant (mean iambic = 0.24, mean trochaic = 0.15, *P* = 0.06).

One bird of the intensity condition also reached a correct proportion >0.60 for three consecutive days and was tested. His responses to the test strings were quite similar to those of the birds in the pitch condition. He responded more with pecks on the trochaic keys in Tests 1 and 2 (1: mean iambic = 0.09, mean trochaic = 0.34, *P* = 0.01; 2: mean iambic = 0.06, mean trochaic = 0.29, *P* = 0.01) and showed no difference between the iambic and trochaic key in Tests 3 and 4 (Test 3: mean iambic = 0.25, mean trochaic = 0.29, *P* = 0.43; Test 4: mean iambic = 0.39, mean trochaic = 0.35, *P* = 0.68).

## Discussion

All zebra finches in this study learned to discriminate between trochees and iambs when the tones varied in pitch. Sequential tests showed that the zebra finches grouped the tones of long alternating strings into smaller sets. Apparently, the birds perceived the strings not as single tones alternating in pitch, but grouped certain tones as if they formed a set. The birds responded more to the long strings as being similar to the trochees than to the iambs of the training, which shows a trochaic grouping of pitch-alternating tones, similar to humans. The zebra finches did not have a grouping bias for strings with a repetition of one tone, without alternations in pitch. In comparable tasks, humans do group non-alternating tone strings as containing iambs or trochees (Hay and Diehl 2007). Moreover, when the tones varied in duration or intensity, only one zebra finch per group learned to distinguish trochees from iambs within 20,000 trials. The results of the bird in the intensity condition closely matched those of the birds in the pitch condition; he perceived long strings with known tones or tones within the training range as trochees. These results are in line with findings in human adults and infants, in which the participants also group alternations in intensity as trochees, independent on acoustic experience (Iversen et al. 2008; Yoshida et al. 2010). The bird in the duration condition showed contradictory results; he showed trochaic grouping for alternations in duration with known tones or tones within the training range. However, he showed an iambic grouping for alternations in duration of tones outside his training range, allowing no clear conclusion. For both birds, it should be noted that they did not reach the standard discrimination criterion, but only had a moderate ability to discriminate iambs from trochees. The difficulty that the zebra finches showed in discriminating the sequences consisting of notes varying in duration is surprising, as zebra finches that were trained in a similar set-up to discriminate between tonal strings with a regular and an irregular beat pattern were able to learn this discrimination by using the exact duration of the tones and the length of the pauses between them (ten Cate et al. 2016). Zebra finches can thus be sensitive to small differences in duration, but most did not use this sensitivity to distinguish iambs from trochees.

The zebra finches did not show perceptual grouping of strings with tones that were outside of their training range. This could be either an effect of the novelty of these tones, or of them being at the limits of the birds’ hearing range. Responding to strings consisting of novel tones on the basis of their pitch pattern requires relative pitch perception, an ability only found in a few bird species (Hulse et al. 1995; Watanabe et al. 2005; Brooks and Cook 2010; Hoeschele et al. 2012), which seems to be especially challenging when the tones are presented sequentially as in the current study (Hoeschele et al. 2015). Apparently, the zebra finches in the current experiment were able to relate the pitch alternations of the new tones within the training range to those of the training stimuli, demonstrating some degree of relative pitch perception. However, the birds may have been unable to use relative pitch to discriminate test strings with pitches outside their training range. The zebra finches also showed reduced responses to these strings and to the strings in which the tones did not alternate. Reduced responses to novel tone strings are found more often with experiments in a go/no-go paradigm (van Heijningen et al. 2013; Chen et al. 2015). It is likely that this is due to an avoidance strategy. There were always known training stimuli presented intermixed with the novel test items, which made it possible to avoid punishment by not responding to the novel items, whilst still receiving food for correct responses to training items. Our results show that the zebra finches in general responded less often when they heard a test stimulus. Moreover, when the tones were outside the trained range or when the tones were not alternating their response rates dropped even further. This shows that these strings were probably considered more novel than the long strings with known tones or tones within the training range.

On the whole, the zebra finches in the pitch condition seem to behave similarly to humans, who also group sound strings alternating in pitch into trochees. Moreover, in humans this grouping bias occurs regardless of the precise nature or familiarity of the sounds (Trainor and Adams 2000; Friederici et al. 2007; Hay and Diehl 2007). For example, Hay and Diehl (2007) tested whether adult listeners responded differently to strings consisting of non-speech tones or of synthetic speech sounds. In their experiments, the (English-speaking) participants grouped strings alternating in intensity or pitch as trochees for both sound types. Other studies used more tone-like sound items (Iversen et al. 2004; Molnar et al. 2014), still finding the same perceptual grouping biases. Furthermore, the bias towards grouping alternations in pitch as trochees is not influenced by the participants’ native language (Iversen et al. 2004, 2008). Lastly, this general grouping bias is not restricted to the acoustic domain but is also found in the visual domain (Peña et al. 2011). For example, when participants saw a string of visual objects that were alternating in flashing rate or in brightness, they grouped them as trochees, perceiving the “stressed” objects with a higher flashing rate or brighter objects as the start of the memorized duplets. These examples suggest that the mechanism underlying the trochaic bias seems to be an experience-independent, universally shared mechanism. Our results strengthen this claim, as we show that the trochaic grouping bias is also present in a non-mammal species, the zebra finch. Like humans, these phylogenetically distant animals group tonal strings with frequency alternations as trochees, suggesting that there might be more ancient evolutionary roots to this perceptual mechanism.

Grouping of alternations in duration, however, is not universal but seems to depend on previous acoustic experience, both in humans (Iversen et al. 2008, Crowhurst and Olivares 2014) and in rats (Toro and Nespor 2015). These experiments showed that infants and animals who show a perceptual grouping bias for pitch- or intensity-alternating sequences do not necessarily also show this bias for duration-alternating sequences. Thus, although both tones alternating in pitch and tones alternating in duration are grouped perceptually, it is likely that these two sound types are processed by different mechanisms, one guided by acoustic experience and the other more universally shared. The zebra finches in the current experiment were mostly unable to discriminate iambic and trochaic stimuli with alternations in intensity or duration, whilst they readily did this with the pitch alternations. Whether the zebra finches lack a perceptual grouping bias for duration and intensity or whether they cannot detect any structure in the short training strings remains unclear. However, the difference in discrimination abilities between the three conditions is an indication that the perceptual mechanisms involved in discriminating alternations in pitch, duration and intensity might also differ in the zebra finch.

Various bird species, like zebra finches and budgerigars, are known to be sensitive to the prosodic features of human speech (Naoi et al. 2012; Spierings and ten Cate 2014; Hoeschele and Fitch 2016). Zebra finches can learn to discriminate between quadruplets of speech syllables with initial or final stress created by increasing the pitch, duration and intensity of a single syllable. This discrimination holds even when only the pitch or the duration cue is increased in the sound. Surprisingly, in the current study the zebra finches were unable to learn to discriminate between tonal quadruplets differing in the ordinal position of long and short tones. It might be that differences in tone durations and intensities are only well perceived by zebra finches when they are accompanied by other prosodic cues. It is unlikely that the differences between the tones in the current study might have been too subtle, since zebra finches have been shown to perceive these differences (Okanoya and Dooling 1990).

To summarize, our study shows that the perceptual bias to group pitch variations into trochees is not specific to humans. After it being shown for rats, we now show that zebra finches share the same perceptual principle. It confirms that this trochaic grouping bias seems independent of linguistic experience and suggests it may be a universal perceptual primitive.

## Appendix

Example of the different test stimuli for each of the three conditions. These examples are based on the training stimuli sets presented in Table 1. Each training set had matching set of test stimuli, based on the same algorithm. All test strings consist of 26 tones in total, each tone separated from the next by a 60-ms pause. Each string started and ended with a fade of 1.3 s. The total duration of the strings in the pitch and amplitude conditions was 3.18 s; in the duration condition, it depended on the duration of the tones in the stimuli and ranged between 3.18 and 8.2 s. In Test 1, the tones are the same as used in the second and third training stimuli. Test 2 uses tones that the birds have not heard in the training, but lie within the range of the training tones. Test 3 also uses new tones, but these are outside of the range of the training tones. The stimuli of Test 4 are a repetition of one tone, and the tones used are from the second and third training stimuli.

**Table:**

(a) Pitch condition	Iambic start (tone frequencies in Hz)	Trochaic start (tone frequencies in Hz)
Test 1—known tones	…1875–2344–1875–2344…	…2344–1875–2344–1875…
	…2344–2930–2344–2930…	…2930–2344–2930–2344…
Test 2—new tones within training range	…2109–2637–2109–2637…	…2637–2109–2637–2109…
Test 3—new tones outside training range	…1200–1500–1200–1500…	…1500–1200–1500–1200…
	…3662–4578–3662–4578…	…4578–3662–4578–3662…
Test 4—repetition of known tones	…1875–1875–1875–1875…	
	…2344–2344–2344–2344…	
	…2930–2930–2930–2930…	

(b) Duration condition	Iambic start (tone durations in ms)	Trochaic start (tone durations in ms)
Test 1—known tones	…60–90–60–90…	…90–60–90–60…
	…90–135–90–135…	…135–90–135–90…
Test 2—new tones within training range	…75–112–75–112…	…112–75–112–75…
Test 3—new tones outside training range	…27–40–27–40…	…40–27–40–27…
	…202–304–202–304…	…304–202–304–202…
Test 4—repetition of known tones	…60–60–60–60…	
	…90–90–90–90…	
	…135–135–135–135…	

(c) Intensity condition	Iambic start (tone amplitudes in dB)	Trochaic start (tone amplitudes in dB)
Test 1—known tones	…52–57–53–57…	…57–53–57–52…
	…57–62–57–62…	…62–57–62–57…
Test 2—new tones within training range	…55–60–55–60…	…60–55–60–55…
Test 3—new tones outside training range	…42–47–42–47…	…47–42–47–42…
	…67–72–67–72…	…72–67–72–67…
Test 4—repetition of known tones	…52–52–52–52…	
	…57–57–57–57…	
	…62–62–62–62…	
